# Hidden Markov Models for Evolution and Comparative Genomics Analysis

**DOI:** 10.1371/journal.pone.0065012

**Published:** 2013-06-07

**Authors:** Nadezda A. Bykova, Alexander V. Favorov, Andrey A. Mironov

**Affiliations:** 1 A.A. Kharkevich Institute for Information Transmission Problems RAS, Moscow, Russia; 2 Department of Bioengineering and Bioinformatics, M.V. Lomonosov Moscow State University, Moscow, Russia; 3 Department of Oncology, Division of Biostatistics and Bioinformatics, Johns Hopkins University School of Medicine, Baltimore, Maryland, United States of America; 4 State Research Institute of Genetics and Selection of Industrial Microorganisms GosNIIGenetika, Moscow, Russia; 5 Vavilov Institute of General Genetics, Russian Academy of Sciences, Moscow, Russia; Hebrew University at Jerusalem, The Alexander Silberman Institute of Life Sciences, Israel

## Abstract

The problem of reconstruction of ancestral states given a phylogeny and data from extant species arises in a wide range of biological studies. The continuous-time Markov model for the discrete states evolution is generally used for the reconstruction of ancestral states. We modify this model to account for a case when the states of the extant species are uncertain. This situation appears, for example, if the states for extant species are predicted by some program and thus are known only with some level of reliability; it is common for bioinformatics field. The main idea is formulation of the problem as a hidden Markov model on a tree (tree HMM, tHMM), where the basic continuous-time Markov model is expanded with the introduction of emission probabilities of observed data (e.g. prediction scores) for each underlying discrete state. Our tHMM decoding algorithm allows us to predict states at the ancestral nodes as well as to refine states at the leaves on the basis of quantitative comparative genomics. The test on the simulated data shows that the tHMM approach applied to the continuous variable reflecting the probabilities of the states (i.e. prediction score) appears to be more accurate then the reconstruction from the discrete states assignment defined by the best score threshold. We provide examples of applying our model to the evolutionary analysis of N-terminal signal peptides and transcription factor binding sites in bacteria. The program is freely available at http://bioinf.fbb.msu.ru/~nadya/tHMM and via web-service at http://bioinf.fbb.msu.ru/treehmmweb.

## Introduction

The task of reconstruction of ancestral states given a phylogeny and discrete states for extant species is known as a common biological challenge. The examined states could be any morphological or behavioral features of the organisms [1]–[4]. In the area of molecular evolution, the problem arises in the context of reconstructing ancestral amino acids at particular sites [5] or gene repertoire in ancestral genomes [6]. The most popular software for this kind of task is BayesTraits (BT) program [5]. It implements the standard Bayesian MCMC analysis applied to the Continuous-time Markov model for the traits evolution [7], [8]. Bayesian inference enables careful handling of the ancestral states uncertainties as compared to parsimony and ML strategies.

In many cases, the problem of phylogenetic uncertainty is relevant. Indeed, the phylogeny is never known exactly, as far as it is reconstructed rather than observed. The BT program solves this by its possibility of taking a set of possible phylogenies as an input; this set is then included into the sampling process as a additional parameter with the flat prior. Another approach to this problem is implemented in the BEAST Software where the joint reconstruction of phylogeny from the sequences and the traits is considered [9], [10].

The model described above does not cover cases when the discrete states are not known for sure. Although similar to the phylogenetic uncertainty, such an uncertainty in the extant states data is also possible. The situation often arises in the field of bioinformatics when, after the computational analysis of genomes, some biological features are predicted. A typical example is an evolutionary analysis of transcriptional regulation: the program predicting the presence or absence of the transcription factor binding site (TFBS) produces a score that reflects a biological state; however, it does not identify precisely the states themselves. The simplest approach to this kind of problem would be to define a score threshold, transform the scores at the leafs into discrete states, and analyze the discrete data. However, even with a perfectly chosen threshold, the scores falling into the (i.e. near threshold) would be, with nearly 50% probability, wrongly transformed into the discrete states. Moreover, the data with mistakes in the assignment of the states to leafs provides significantly worse results (we test it here by simulations). The situation can be improved by smarter models.

In [11], the authors aimed to improve the prediction of transcriptional regulatory networks. They developed an iterative two-step likelihood-maximizing algorithm that used evolutionary information to refine the leaf states.

The Hidden Markov Model (HMM) strategy for this task was originally proposed by [12] in a study of the evolution of CRP binding sites in intergenic regions of *E. coli, S. typhium* and *Y. pseudotuberculosis*. This HMM considered the presence of neutral or negative selection affecting the given locus as a hidden state, and the TF binding energy was the emitted value, which was observed at the leafs of the evolutionary tree. The probability distribution of the TF binding energy was supposed to be known for both hidden states. Transition probabilities between states were identified through simulations of the TFBS energy changing under the two selection modes. In a similar model [13], two modes of sequence character evolution were explicitly used to calculate the transition probabilities between states. Another implementation of this approach was performed by [14] in a study of the evolution of transcriptional regulation in three animal species (human, mice and cow). However, these probabilistic evolutionary models could consider only a few (up to four) species on an evolutionary tree. The application of these approaches for an arbitrary number of species is hindered: the constraint of only one event on a tree used in [12] and [13] is inappropriate for large trees; in a general case [14] the amount of the calculations grows exponentially with the number of species.

**Figure 1:**
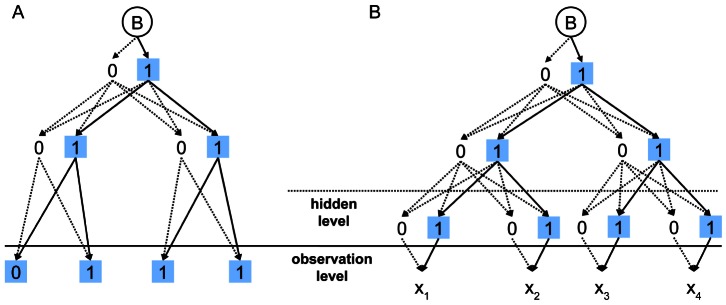
Models for the ancestral state reconstruction. 0 and 1 are two possible states at the tree nodes. The solid edges reflect transitions for the optimal states assignment; the dotted edges are non-optimal edges. The blue boxes denote optimal states at the nodes. B is the start point. (**a**) The discrete state model with observable states at the leaves; (**b**) the HMM model with observable scores and a hidden layer of states at the leaves. Note that in (b) optimal states at the leaves are chosen from the full set of states, while in (a) they are fixed to observable states.

Here we represent our novel, unified tHMM approach that combines the ancestral state reconstruction and the statistical prediction of the leaf states. tHMM intends to simultaneously reconstruct the leaf states, the ancestral node states and the evolution rates given the observed scores at the leafs and the phylogeny. The core of the approach is a HMM model on the evolutionary tree [12], [14], which is, in turn, a special case of Bayesian networks [15], where the hidden states at leaves underly the observed score values (Fig. 1). An important feature of the suggested model is its applicability for large tree problems.

Below we provide (1) the description of the tHMM model and the algorithms for states reconstruction, (2) simulations showing tHMM’s advantage over the BayesTraits reconstruction from the corresponding discrete states, (3) two examples of applying the model to real biological systems, (4) the standalone program and the web service implementation of the algorithms.

## Materials and Methods

### Evolutionary model

#### Continuous-time Markov model of discrete trait evolution

A continuous-time Markov model of trait evolution consists of a set of possible states and transition probabilities between these states per a unit of time. For the case of two states, the probability of the state transition in time 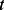 (or tree branch length) can be written as
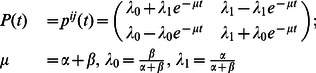
(1)where 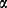 and 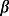 are the transition rates for 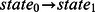 and 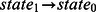, respectively [16].

The evolutionary processes on every branch of the tree are considered to be independent. This assumption allows easy calculation of the probability of observing a given set of states at the leaves of the evolutionary tree
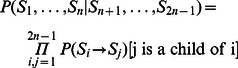
(2)where 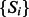 is the set of states at tree nodes; the leaves are enumerated from 1 to 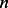, the ancestral nodes – from 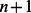 to 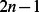; 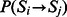 is the transition probability from state 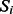 to state 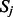; and z is notation for the indicator function that is 1 if the predicate is true, or 0 otherwise [P1].

In this model the input values are states at the leaves; if these states were defined from the prediction scores 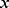 with the threshold 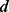, the states 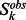 are defined as
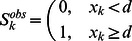



Within the specified model, the ancestral states probabilities can be evaluated given observed states at the leaves [5], [7]. The program mode that implements only this model will be referred to as dumbtHMM mode. It is useful for the data with discrete states assigned to leaves.

#### tHMM modification for hidden states

In our model, in contrast to the standard one described above, the input values are prediction scores; therefore, to calculate the probability of the input scores, the emission probabilities of scores for each state should be additionally defined. At this point our model becomes a Hidden Markov Model, as we observe data generated by underlying unobservable states. A schematic illustration of the standard problem of ancestral state reconstruction and our modification is shown in Fig. 1.

When the states at all nodes are defined, the probability of the observed scores can be calculated as
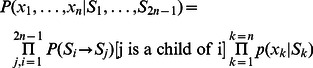
(3)where 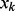 are the predicted scores at the leaves, 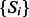 is the set of states at tree nodes (including the terminal ones), 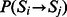 is the transition probability from state 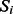 to state 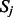, and 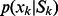 is the emission probability of 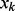 for state 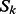.

The total probability to observe the data is the sum of probabilities under all possible sets of states at nodes:
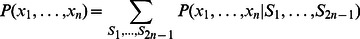
(4)


Usually, the observed scores are values of a continuous random variable. In this case the emission probabilities are given as probability densities; hence, the probabilities in equations (3–4) should also be considered as probability densities.

Now we can define the problem.

**Given:**

- An evolution tree
- Score observations at the leaves
- Prior probability distribution of scores for states 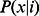

**Get:**

- States at all nodes of the tree including the leaves.

In the HMM theory, the general decoding problem can be formulated in two different forms: to find the state assignment that maximizes the probability to observe the data, and to find the posterior probabilities for states at each node. The first approach yields the Viterbi algorithm; the second one, the posterior decoding algorithm.

### Algorithms for the Reconstruction of the Node States

#### The Viterbi algorithm

In the common maximum likelihood approach, the desired set of states is the one that maximizes the probability of the observed data. For our model, the likelihood function is given by equation (3), and the ML estimate can be found by a modification of the Viterbi algorithm. The Viterbi variables 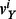 for each node 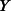 and state 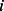 correspond to the maximum probability of the data on the leaves of the subtree starting at the node 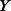 at the state 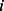. The transition probabilities from the start point 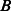 are the prior probabilities 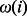 for the states. The Viterbi recursion for this case can be written as
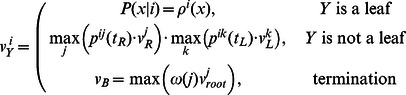
(5)

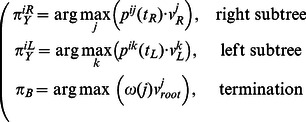
(6)


Here superscripts indicate states, subscripts indicate tree nodes; 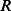 is the right child node, 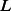 is the left child node; 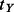 is the distance from the node 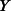 to the parent node 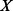, 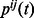 is the transition probability from the state 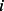 to the state 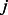 in time 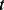 defined by equation (1), 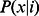 is the probability of observing 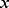 in the state 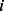, 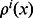 is the score probability distribution for the state 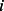. The traceback variables 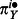 store transitions from the state 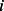 at the node 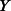 to the child node that provided the 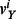 value. 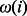 is the prior probability of the state at the root. This probability can be defined as the equilibrium probability 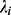 defined by equation (1).

The recursion starts from leafs and propagates to the root. When the upward process is finished, the variables 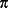 are used to reconstruct the states in the reverse passage to the leafs.

#### Posterior decoding. Up-down algorithm

Similarly, to infer the states probabilities at nodes, the forward-backward posterior decoding algorithm can be modified for a tree. We call this algorithm the *Up-Down* algorithm.

**Figure 2:**
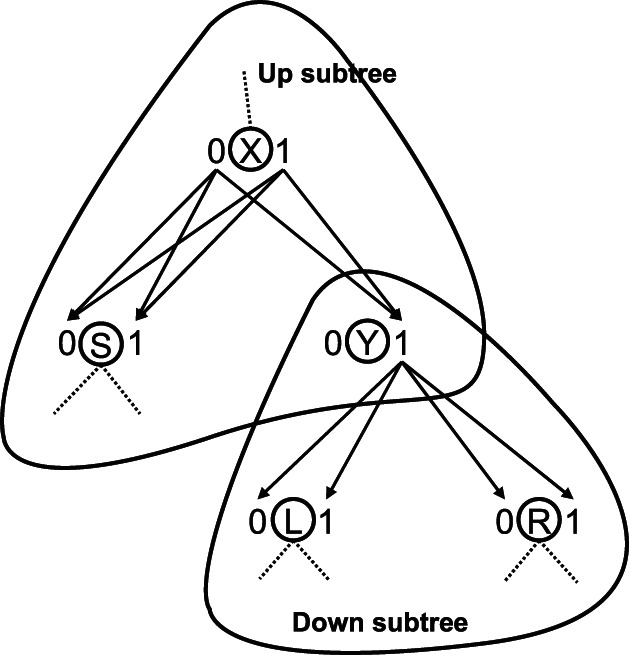
The Up-Down algorithm. Partitioning of a tree relative to state 1 at the node 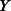 is shown by dashed lines.

The probability of a state 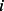 at a node 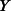 is the ratio of the overall probability of state sets with the fixed state 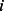 at the node 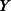 to the total observation probability:
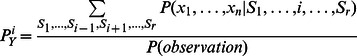



The overall probability of sets having the state 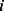 at the node 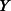 can be written as a product of two factors: the probability of the subtree where the node 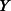 is the root (the bottom tree in Fig. 2; 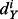) given the state 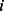 at this root, and the probability of the subtree where the node 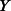 is a leaf (the top tree in Fig. 2; 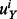) given the state 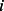 at this leaf :
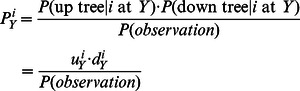
(7)


The *up* and *down* variables can be calculated recursively.
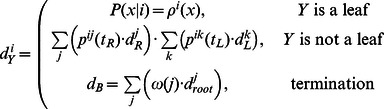
(8)

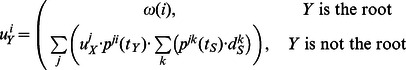
(9)


Here 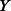 is the current node; 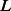 and 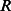 are the left and right child nodes; 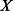 is the parent node; 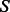 is the sister node. The *down* variables 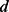 are calculated upward from the leafs to the root; the *up* variables are calculated downwards from the root to the leafs. The total probability of an observation is
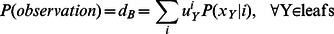



The posterior decoding approach allows for prediction of the probabilities of states on the nodes as well as evaluation of the probabilities of state transitions, i.e. the probabilities of evolutionary events. The posterior probability of an evolutionary event on a branch 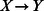 of the evolutionary tree can be calculated using the equation
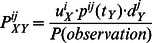
(10)


The described algorithms generalize the existing algorithms for the case with uncertain states at the leaves. Our variant of the Viterbi algorithm is a direct analogue of the weighted parsimony method [17] used for the standard problem, where the leaves states are assigned with certainty. Similarly, an analogue of the upward part (8) of the Up-Down algorithm was described by Felsenstein as a pruning algorithm [18].

### The Parameters Evaluation

The algorithms (5, 8, 9) depend on the following parameters: (a) the prior score distributions 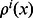; (b) the transition rate parameters 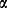, 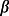 in equation (1). The first parameter reflects *a priori* knowledge about the biological problem and will be discussed later. The transition rates can be estimated by the standard likelihood maximization (ML) approach. Here we used the Bayesian inference of the posterior probability distribution of model parameters using Metropolis-Hastings MCMC (Markov Chain Monte Carlo) sampling technique, following [5].

### The Dataset

#### Signal peptides data set

Protein sequences of Gram-negative bacteria were downloaded from GeneBank release 175 (ftp://ftp.ncbi.nih.gov/genomes/Bacteria/). Orthologous protein groups were downloaded from the NCBI Protein Clusters database [19] release Jan 2010 (ftp://ftp.ncbi.nih.gov/genomes/CLUSTERS/). The data set contained 717455 proteins from 593 genomes. Multiple alignments were constructed by Muscle [20]. Protein phylogenetic trees were created using the protdist and neighbor programs in the PHYLIP package [21]. Signal peptide scores were calculated by SingalP 3.0-NN [22]. In the evolutionary analysis, we considered a subset of orthologous clusters where different discrete predictions of signal peptides were present.

#### CRP transcription factor binding sites data set

Orthologous groups and the phylogenetic trees were downloaded from the MicrobesOnline resource [23]. Transcription factor binding scores were downloaded from the RegPrecize database [24].

## Results

### Simulations

To test the ability of tHMM to improve the state reconstruction accuracy and to define the range of tHMM applicability, computer simulations were performed. We compared the efficacy of tHMM itself, the dumbtHMM and the BayesTraits Mulitstate software on a set of different simulated datasets.

The simulation parameters were rates of the state changes (transition rates) and the score distributions for states. 16 sets of simulated data (phylogeny and scores at the leaves) were generated (see Text S1), each consisting of 400 trees.

Phylogenies were generated by sampling the branch lengths from the distribution that was obtained from the Signal peptides dataset. The speciation process was terminated and a leaf was created if the distance from the root to the current node exceeded 0.5. This constraint restricted the tree sizes to the range of 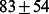 leaves. For each new node, one of the two states was assigned according to its parent node state and to the transitions probabilities from Eq. (1).

The score distributions that modeled the outer prediction program were chosen to be 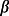-distributions, and the leaf scores were sampled from the corresponding score distribution (see Text S1, Fig. S1). S1. For the BayesTraits and dumbtHMM model, the discrete state mapping for the leaves was defined with a score threshold derived from the respective (as in simulations) score distributions to satisfy the rule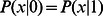. For the tHMM, scores themselves were taken as an input, and the same score distributions as in simulations were used, as if they were known or inferred with perfect precision. This situation, of course, never happens in real life, but it was done to infer the maximum improvement that can be achieved by using the new model.

For each tree, the reconstruction procedures by the three algorithms were run and the results were aggregated in each dataset into the following characteristics: The nodes accuracy was calculated as the proportion of correctly restored states. The overall number of events was normalized by the sum of all the tree lengths to monitor the number of restored events. The Matthews correlation coefficient (MCC) [25] characterists were calculated for the reconstruction of the state change events.

**Figure 3:**
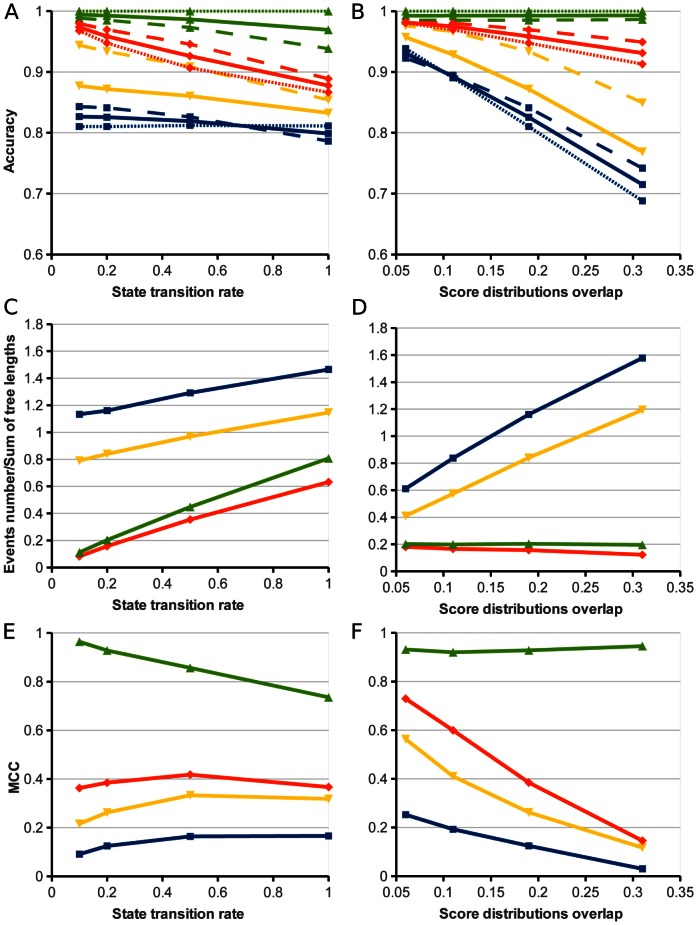
The comparison of the tHMM, dumbtHMM and BayesTraits on simulated data sets. (**a**) The nodes, leaves and total accuracy of states reconstruction for simulations with varying transition rates and with fixed score distributions overlap value = 0.19. (**b**) The nodes, leaves and total accuracy of states reconstruction for fixed transition rate = 0.2 and varying score distributions overlap values. The red lines represent the tHMM results; the blue lines, BayesTraits results; the yellow lines, the results of dumbtHMM; and the green lines represent the results of the dumbtHMM reconstruction from the known assignment of states to leaves. The dashed lines show the accuracy for internal nodes reconstruction; the dotted line, the accuracy of leaves assignment (the yellow dotted line coincides with the blue dotted line); and the solid line, the mixed accuracy for all the nodes of the tree. (**c**) The number of reconstructed events normalized by the total tree length in the set for the same settings as in (**a**). (**d**) The number of reconstructed events normalized by the total tree length in the set for the settings as in (**b**). In (**c**) and (**d**), the green line represents the real number of events; the blue line, the number of events reconstructed by the BayesTraits algorithm; the red line, by the tHMM algorithm; and the yellow line, by the dumbtHMM algorithm. (**e**) The Matthews correlation coefficient(MCC) for the accuracy of events reconstruction for the same settings as in (**a**). (**f**) The MCC for the accuracy of events reconstruction number of reconstructed events for the settings as in (**b**). In (**e**) and (**f**), the green line represents the results of the dumbtHMM reconstruction from the known assignment of states to leaves; the blue line, the results of the BayesTraits algorithm; the red line, by the tHMM algorithm; and the yellow line, by the dumbtHMM algortihm.

The results of the simulations are shown an Figure 3. First,the accuracy of the tHMM prediction of states outperforms other option for both inner nodes and leaves (panels **(a)** and **(b)**). Second, discrete methods dramatically overpredict the number of events but tHMM does not (panels **(c)** and **(d)**). Third,a tHMM reconstructs the events on the tree better than the discrete methods (panels **(e)** and **(f)**). Nevertheless, it appears **(f)** to be useless to apply any of these methods to the data with 1/4 or more percents of mistakes in the leaves assignment.

The possible reasons why the BayesTraits algorithm performs worse even than discrete dumbtHMM is considered in Discussion.

### Signal Peptides

**Figure 4:**
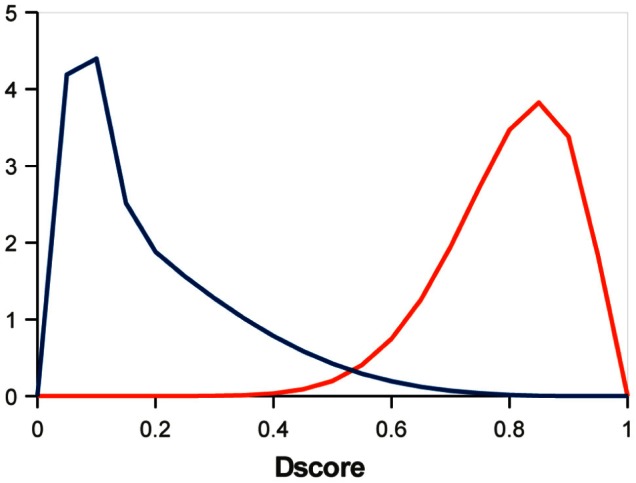
Score distributions for states. The blue line corresponds to the SignalP Dscore distribution of the 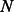 state (no signal peptide); the red one, to the distribution of the 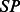 state (signal peptide present).

Bacterial signal peptides are 15–30 aa sequences at the N-terminus of a protein that direct it to the export from the cytoplasm [26]. Here we consider Sec-type signal peptides, which can be predicted by the SignalP 3.0 program [22], [27]. We used a simple model having two biological states: state 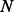 corresponding to the signal peptide absence, and state 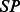 to the signal peptide presence. The observed values were theDscores predictions of SingalP3.0-NN. To determine the score distributions for the states, we represented the Dscore distribution on the entire dataset as a weighted mixture of two beta distributions (Text S2, Fig. S2). One of these distributions was assigned to the 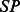 state, the other to the 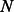 state (Fig. 4).

For the the discrete case we assigned the DScore threshold to the value 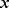 satisfying 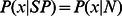 instead of using the default SignalP value.

The event statistics for the whole dataset,shown in Table 1, show that the number of reconstructed events is 2.5-fold lower than for the parsimony. It illustrates the filtering out of the noise events.

**Table 1:** Events statistic for the both data sets.

	Signal data set		TFBS data set	
	parsimony	tHMM	parsimony	tHMM
Number of events	7015	2778	16854	3984
Number of gain events	–	1253	–	2678
Number of loss events	–	1525	–	1306
Clusters with events	2588	1010	1977	1160

The comparison of the distribution of posterior and prior probabilities of states at the leaves over all the data set shows a significant decrease in the number of predictions with state N probability that are in the interval [0.4; 0.6], i.e., that are in the ‘grey zone’ (Table 2).

**Table 2:** The number of entries in the grey zone for both data sets.

	all entries	prior probability in [0.4;0.6]	posterior probability in [0.4;0.6]
Signal peptides	133345	5207	1207
TFBS	307903	13005	1788

**Figure 5:**
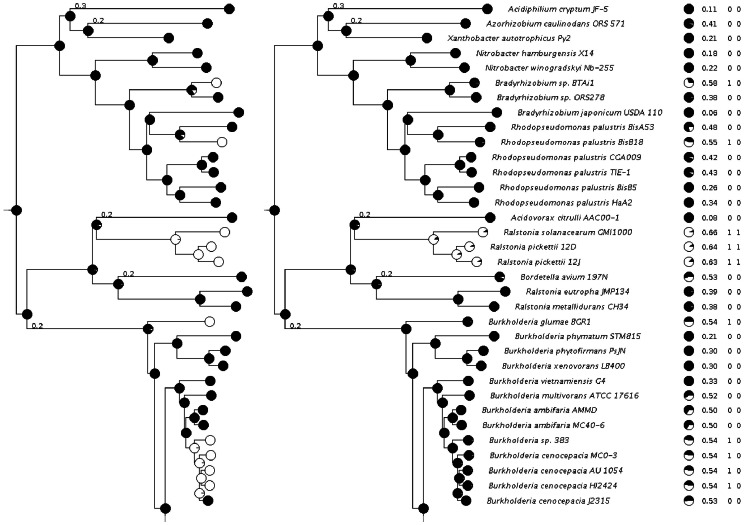
The results of the dumbtHMM (left) and tHMM (right) algorithms applied to the signal peptide reconstruction at the amidase othologous cluster (PRK07056) tree. The black segment of a circle reflects the posterior probability of state 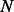 (non-signal) at a particular node. The column with circles at the right shows the prior probability of state 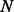 at the leaves, calculated from the score distributions. The remaining columns left to right: Dscore, prior state, posterior state from the tHMM algorithm at the leaves.

Figure 5 shows the results of the tHMM and dumbtHMM methods applied to the amidase orthologous protein cluster (PRK07056). The dumbtHMM algorithm reconstructs six evolutionary events, five gains and one loss of a signal peptide, while tHMM yields only one loss event, with seven prediction corrections at leaves. The ancestral node reconstructions differ only at the *Burkholderia* nodes.

As an additional argument in favor of the tHMM reconstruction, sequence alignments of the branches where algorithms differ in their predictions may be considered (Fig. S3). At the N-terminus of the *Burkholderia* branch alignment, where tHMM rejected several events, the Dscore fluctuations near the decision threshold were produced by small sequence changes and are unlikely to be the reason for several biological state changes during such a short period of time. An opposite situation occurs at the *Ralstonia* branch where tHMM confirmed an event: there is no good N-terminal alignment with the closest neighbors, but, due to large scores and relatively long branch length,this event was accepted.

### TFBS

The binding motif of the CRP transcription factor is known to have low specificity and when applied to genomic data produces numerous false positives [28], [29]. On the other hand, some experimentally determined sites have low scores [30]. Applying an evolutionary probabilistic model could help to increase the prediction quality and simultaneously identify the gain/loss events.

The prediction score for a gene was defined as the best PWM score in the gene’s upstream region. The background score distribution was defined for each gene separately as a distribution of the maximum score on a random sequence whose length equaled the length of the gene’s upstream region, whereas the positive distribution was the same for all species and genes and was set to the normal approximation of positive experimental data scores.

A brief summary of the reconstructed events is presented in Tables 1 and 2. For the TFBS case, an even stronger decrease in events number (four-fold) is observed, which is in line with a weaker accuracy of the TFBS predictions compared to the signal peptides predictions.

**Figure 6:**
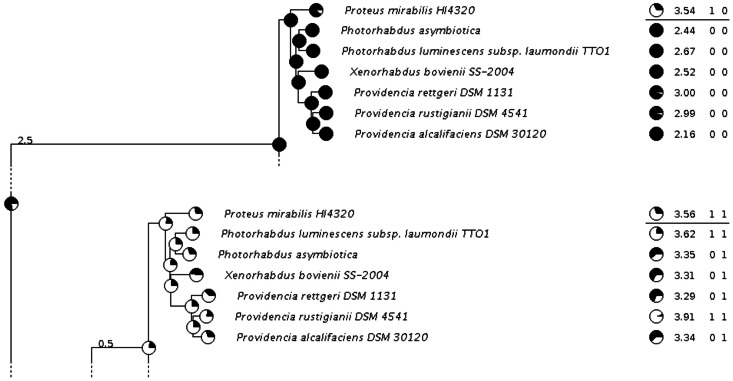
The results of tHMM for the TFBS reconstruction of the AsnB (L-asparaginase) tree. Notation as in Fig. 5. The score values are Z-scores.

Figure 6 shows the results for the AsnB (L-asparaginase) group of orthologs. The example was selected to demonstrate the tHMM power to account for the evolutionary context of predictions. Here, the *Proteus mirabilis* HI4320 paralogs have close scores, 3.54 and 3.56, but, in different evolutionary contexts, different underlying states are inferred. It is clear that a pair of nearly equal scores from a middle-value interval can easily be produced by different underlying states. Such cases can not be properly resolved using threshold-based methods. The tHMM approach allows for the inference of different underlying states for the same score depending on the context.

### Program

The tHMM program is available as a standalone program at http://bioinf.fbb.msu.ru/~nadya/tHMM and as a web service at http://bioinf.fbb.msu.ru/treehmmweb. All userguide instructions and format requirements are present at the specified resource.

## Discussion

Here we present and analyze a novel tHMM approach for reconstruction of states of a known tree. The approach explores all possible combinations of the states on the leaves in a Bayesian way. The phylogeny consistency analysis provides the prior; and the correspondence of hidden leaf states to the observed score provides the likelihood for each combination.

The method allows reconstruction of evolutionary events and states in the tree nodes from the prediction scores. The method takes into account the prediction program accuracy and does not overestimate the rate parameters and the number of events. It is especially significant when the feature prediction program has low accuracy, and a large part of the observed scores belongs to a gray zone. However, there is the risk of losing very recent events.

In the present work, we used the tHMM version that works with a defined tree to demonstrate the advantages of the method on a basic case, although. the Bayesian approach we used can incorporate the phylogenetic uncertianity in a common way [5].

During the simulation, we found that the BayesTraits algorithm performs worse than discrete dumbtHMM (see Figure 3). However, the overall tree likelihood for these two methods was exactly the same for each tree (not shown). The difference is probably because BayesTraits reconstructs the node states only from the node’s subtree while dumbtHMM (as well as tHMM) provides forward-backward analysis for this task.

## Supporting Information

Figure S1 **Score distributions for states used in simulations.** Distribution for state 0 is at the left; for state 1, at the right.
(EPS)

Figure S2 **Dscore histogram approximation.** **a)** Approximation of the Dscore histogram (red circles) by a weighted sum of Beta distributions (black). **b)** Approximation of extra counts in the high Dscore area (red circles) by a Beta distribution (black).
(EPS)

Figure S3 **N-terminus of (A)** ***Burkholderia*** **and (B)** ***Ralstonia*** **nodes alignment.** Signal peptides predicted by SignalP 3.0. are shown in red. Pink denotes signal peptides with Dscore lower than the threshold.
(EPS)

Text S1 **Data simulation parameters.**
(PDF)

Text S2 **Reconstructing score distributions for Signal peptides dataset.**
(PDF)
