# Supplementary material for: Hidden Markov Models for Evolution and Comparative Genomics Analysis
Source: PLoS One. 2013 Jun 7;8(6):e65012. doi: 10.1371/journal.pone.0065012 (PMC3676395; doi:10.1371/journal.pone.0065012)
Supplement: Text S1 — Data simulation parameters. (PDF) [file pone.0065012.s004.pdf]

Supplementary Text 1

Plain simulations parameters

Tree sets of size 400 were generated for different transition rates and score distribution parameters. For a simplicity of graphs we considered equal gain and loss transition rates in the simulation model ($\alpha = \beta$), so that considered rates were $\{0.1, 0.2, 0.5, 1\}$. Pairs of mirror symmetric score distributions (for two states) used in the simulations are shown at Figure S1. The distribution pairs were sorted by their overlap measure. This measure was defined simply as a proportion of area after the point of intersection, reflecting sensitivity and specificity of a hypothetical prediction program. The values of overlap measure were $\{0.31, 0.19, 0.11, 0.06\}$. For the all three reconstruction we used flat prior distribution for transitions rates on the interval (0,2) for both rates. The length of MCMC was 500000, the start point of the sample was 50000, and the sample step, 300.

Real data analysis parameters

In our calculations for both signal peptide and TFBS examples, the MCMC parameters were set as follows: chain length = 10^6 , sample start position = 50000, sample step = 300.
