# Supplementary material for: Hidden Markov Models for Evolution and Comparative Genomics Analysis
Source: PLoS One. 2013 Jun 7;8(6):e65012. doi: 10.1371/journal.pone.0065012 (PMC3676395; doi:10.1371/journal.pone.0065012)
Supplement: Text S2 — Reconstructing score distributions for Signal peptides dataset. (PDF) [file pone.0065012.s005.pdf]

Supplementary Text 2. Reconstructing score distributions for Signal peptides dataset.

The SignalP program produces a DScore value that reflects a propensity of the protein to have a signal peptide. We suppose our protein data set to be a mixture of proteins of two types (states) - proteins with signal peptides (state SP) and without signal peptides (state N). According to this, the Dscore histogram can be represented as a weighted sum of two Dscore distributions corresponding to different states. An approximation of the histogram with a weighted sum of distributions can provide an estimation of score distributions for the states.

SignalP prediction Dscore vary from 0 to 1. So, we decided to search for a solution in the form of mixture of beta distributions. We performed the approximations with R nlm program.

Despite the fact that the histogram is bimodal, the approximation with a mixture of two beta distributions does not detect the rightmost peak (both of the distributions peaks are on the left to the histogram sag, (Fig. S4a)). Only the approximation with a sum of three beta distribution detects it.

However, the contributions of two protein types to histogram differ greatly and the direct approximation of histogram with the sum of three beta distributions is expected to bias the accuracy of reconstructed distribution after normalizing coefficients. So, we used the first two beta approximation as an estimation of the N (no signal peptide) state (Fig. S4a) and then approximated the subtraction of this [N state] approximation from the histogram with the single beta distribution (Fig. S4b).

The resulting score distributions for states are: for the N state $\rho^N(x) = 0.22 * \beta(6.27; 67.18; x) + 0.78 * \beta(1.33; 4.78; x)$, for the state SP $\rho^{SP}(x) = \beta(10.2; 2.6; x)$ (Fig. 4 in the main text).

This is not in a good consistence with the SignalP 3.0.-NN Dscore threshold: it equals to 0.44 for Gram-negative bacetria, while the point of intersection of our distributions is 0.54. This is probably due to the fact, that the SignalP training set poorly represents the general population of studied proteins. Indeed, the SignalP 3.0. negative training set (http://www.cbs.dtu.dk/ftp/hnielsen/GRAM-CYT.reduc.gz) contains only ~300 cytosolic proteins, in contrast to hundreds of thousand proteins in our data set.
